# Epigenetic Mechanisms Underlying Cognitive Dysfunction in Parkinson's Disease: Current Evidence and Future Prospects

**DOI:** 10.1002/brb3.71602

**Published:** 2026-07-29

**Authors:** Fatemeh Hasani, Mohammad Ebrahim Kherad, Mohammad Sharifi Sarasyabi, Kimia Jazi, Ali Ashkbari, Payam Ahmadi, Hadise Heidarpour, Mahdi Masrour, Sina Khamaki, Rahem Rahmati, Andrei Surguchov

**Affiliations:** ^1^ Neuroscience Research Center Golestan University of Medical Sciences Gorgan Iran; ^2^ Gastroenterology and Hepatology Research Center Golestan University of Medical Sciences Gorgan Iran; ^3^ School of Medicine Kerman University of Medical Sciences Kerman Iran; ^4^ School of Medicine Shahid Beheshti University of Medical Sciences Tehran Iran; ^5^ Faculty of Medicine Mashhad University of Medical Sciences Mashhad Iran; ^6^ School of Medicine Tehran University of Medical Sciences Tehran Iran; ^7^ Students Research Committee Shahrekord University of Medical Sciences Shahrekord Iran; ^8^ Department of Neurology Kansas University Medical Center Kansas City Kansas USA

**Keywords:** alpha‐synuclein, cognition, DNA methylation, epigenetics, miRNA, Parkinson's disease

## Abstract

**Purpose:**

Epigenetics studies inheritable characteristics and lasting cellular changes that occur without alterations in the DNA sequence. This field is crucial for understanding how environmental factors interact with genes to influence memory, learning, and cognition. In the context of neurodegenerative disorders, particularly Parkinson's disease (PD), epigenetic mechanisms may help explain the molecular basis of cognitive impairment. The purpose of this review is to explore how epigenetic alterations affect gene expression and their potential role in cognitive dysfunction associated with PD.

**Method:**

This review synthesizes findings from existing literature on epigenetic mechanisms—including microRNA (miRNA) regulation, histone modification, and DNA methylation—in relation to neuronal function and cognitive processes in PD. Relevant studies were identified and analyzed to determine how these mechanisms may contribute to neuroinflammation, synaptic remodeling, and neuroprotection in PD.

**Findings:**

Epigenetic modifications can activate or silence gene expression by altering chromatin structure and protein interactions without changing the DNA sequence. In PD, such changes may influence genes involved in neuroinflammation, neuronal plasticity, and protective signaling pathways. Evidence suggests that dysregulation of DNA methylation, histone modification, and miRNA expression could be linked to the cognitive deficits observed in PD patients. However, the current body of research remains limited and heterogeneous.

**Conclusion:**

Epigenetic regulation represents a promising frontier for understanding cognitive impairment in PD. These mechanisms offer insights into how environmental and molecular factors interact to drive neurodegeneration. Further studies are needed to clarify the specific epigenetic alterations involved and to determine their potential as biomarkers or therapeutic targets for cognitive dysfunction in PD.

## Graphical abstract

Epigenetic mechanismsincluding DNA methylation, histone modifications, and microRNA (miRNA) regulationmodulate gene expression without altering the DNA sequence and are increasingly implicated in the cognitive impairment associated with Parkinson's disease (PD). Environmental and molecular factors influence these epigenetic pathways, leading to altered chromatin dynamics, dysregulated neuronal gene expression, neuroinflammation, impaired synaptic plasticity, and reduced neuroprotection, ultimately contributing to cognitive decline. Understanding these interconnected mechanisms may reveal novel biomarkers and therapeutic targets for preventing or mitigating cognitive dysfunction in PD.

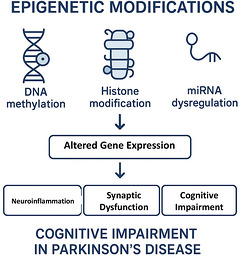

**Abbreviations**
α‐Syn: α‐Synuclein
AD: Alzheimer's disease
BDNF: brain‐derived neurotrophic factor
CPB: cardiopulmonary bypass
HAT: histone acetyltransferase
HDAC: histone deacetylase
HLA‐DP‐1: human leukocyte antigen DP‐1
LB: Lewy body
LPS: lipopolysaccharide
PD: Parkinson's disease
PND: perioperative neurocognitive disorder
POCD: postoperative cognitive dysfunction
PTM: posttranslational modification
ROS: reactive oxygen species
SNpc: substantia nigra pars compacta

## 1 Introduction

As a fast‐advancing field, epigenetics focuses on the interaction between environmental stimuli and gene regulation mechanisms (Yi 2024). These complex mechanisms are crucial for development and maintaining homeostasis. Organisms utilize epigenetic modifications as functional adaptations that help handle and respond to environmental changes (Moosavi and Motevalizadeh Ardekani 2016). Epigenetic mechanisms involve structural adjustments in DNA and related proteins that do not directly modify the underlying DNA sequence. Feedback mechanisms can affect these changes that can regulate the activation or suppression of gene expression (Huynh and Casaccia 2013).

Studying epigenetics and its intricate molecular mechanisms has the potential to uncover the underlying factors responsible for many diseases like cancer, autoimmune disorders, neurodegenerative conditions, and psychiatric illnesses (Zoghbi and Beaudet 2016). This knowledge can serve as a foundation for designing better‐targeted and more potent treatments against these diseases. This review focuses on cognitive dysfunction, particularly in relation to PD. Among neurodegenerative disorders, PD ranks second in prevalence after Alzheimer's disease (AD), and is identified by bradykinesia, unstable posture and gait, hypomuscular rigidity, resting tremor, and kinetic movement disorder (Doder et al. 1999; Goetz 2011).

PD, whether sporadic or familial, is defined by a continuous depletion of dopaminergic neurons in the substantia nigra pars compacta (SNpc) region, potentially caused by the accumulation of protein aggregates known as Lewy bodies (LBs) that contain α‐Synuclein (α‐Syn) (Nair and Ge 2016). However, the specific function of α‐Syn in disease progression remains unclear; it is affected by various genetic and molecular factors, as well as environmental conditions that contribute to α‐Syn accumulation (Cannon and Greenamyre 2013).

Several investigations have focused on the influence of epigenetics on cognition and PD; however, research connecting these findings is limited. The current investigation seeks to evaluate the epigenetic modifications potential impact on expression of genes associated with protecting neurons, inflammation, and synaptic adaptability as possible contributors to the impairments in cognitive function observed in PD. Our aim is to elucidate PD's epigenetic regulatory mechanisms, aiming to clarify the emergence of cognitive impairments associated with this condition and to facilitate designing innovative therapies that focus on modifying epigenetic modifications.

## 2 Parkinson's Disease

**FIGURE 1:**
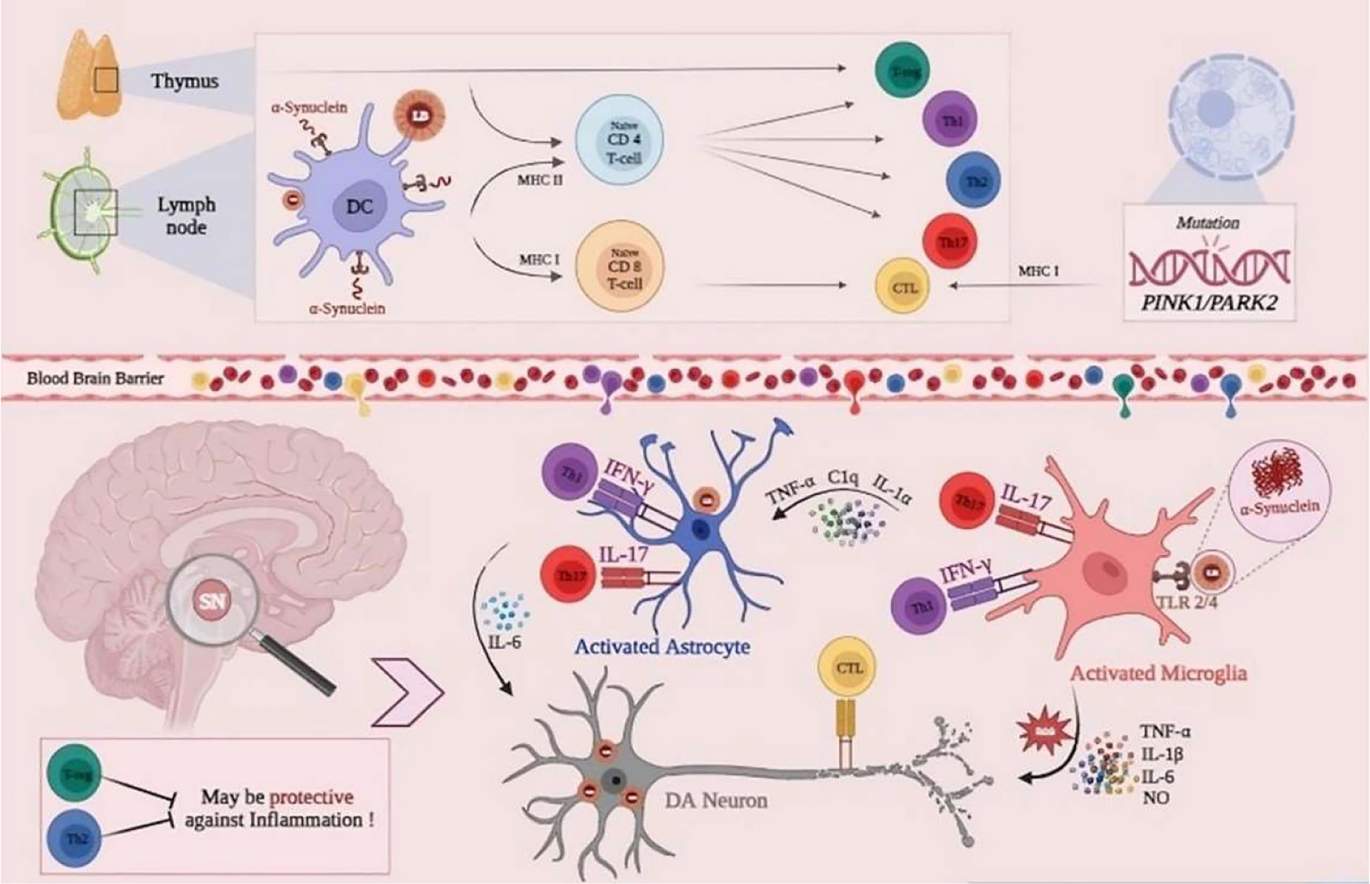
Main players in PD pathophysiology.

Figure 1 illustrates key regulators involved in the pathophysiological mechanisms of Parkinson's disease (PD). There are two types of occurrences for this neurodegenerative disease: familial and sporadic. Genetic abnormalities, including α‐Syn gene mutations, such as *H50Q* (Appel‐Cresswell et al. 2013), *G51D* (Lesage et al. 2013), *A53T* (Polymeropoulos et al. 1997), *A30P* (Krüger et al. 1998), *E46K* (Zarranz et al. 2004), and locus duplication (Nishioka et al. 2006) or triplication (Farrer et al. 2004), contribute to the familial form. The underlying factors behind sporadic PD remain partially unclear, although ongoing research implicates the combination of environmental and genetic factors. Certain pesticides, such as paraquat and rotenone (Tanner et al. 2011), along with the toxin MPTP (1‐methyl‐4‐phenyl‐1,2,3,6‐tetrahydropyridine) (Langston et al. 1983)—a metabolic derivative of the synthetic opioid desmethylprodine (MPPP)—have been recognized as possible cause of sporadic PD. A review conducted by Pan‐Montojo and Reichmann (2014) emphasizes the significant function of toxic environmental agents in the development of sporadic PD. While the precise impact of genetic and environmental factors on sporadic PD is still uncertain, aspects of disease progression such as oxidative stress, neuroinflammation, and the aggregation and misfolding of α‐Synuclein have been recognized (Lema Tomé et al. 2013; Clairembault et al. 2015; Niranjan 2014; Zaltieri et al. 2015)

The misfolding and accumulation of α‐Synuclein are believed to contribute to Lewy pathology in surviving neurons, suggesting that targeting α‐Syn aggregation could have therapeutic potential (Dehay et al. 2015). Sporadic PD probably results from a multifactorial interplay of environmental triggers and genetic factors, prompting ongoing research to identify potential environmental contributors to the disease.

LBs represent a distinctive neuropathological feature of PD, characterized by neuronal protein aggregates. LBs are formed from a combination of vesicular membrane components and abnormal organelles, along with protein accumulation, where α‐Syn is the principal constituent (Spillantini et al. 1997; Shahmoradian et al. 2019). Initial assumptions linking the loss of dopamine (DA) neurons and LBs to the cause of PD have faced scrutiny. Studies by Gibb and Lees (1988) indicated an increase associated with aging in LB prevalence, surpassing PD rates by three‐ to sixfold. Moreover, some confirmed cases of LB pathology show no clinical symptoms, challenging its significance (Parkkinen et al. 2008). Recent research has revealed a lack of complete correlation between cell death and LB presence in affected brain regions. Notably, areas with neuronal death in PD may lack LBs, and vice versa. In patients without dementia, significant neuronal loss occurs in specific cortical regions unrelated to LB presence, questioning the presumed causal link between LBs and cell death in PD (Pedersen et al. 2005; MacDonald and Halliday 2002; Ansorge et al. 1997)

Contrary to assertions that neuronal loss primarily occurs in brain regions with LBs (Halliday et al. 1990; Kremer and Bots 1993), in a study by Iacono et al. (2015) no meaningful relationship was observed between neuronal loss and LBs in PD brains. Furthermore, cases with genetic mutations in PD exhibit distinct LB distributions compared to idiopathic PD. For instance, PD patients with a mutation of the *G2019S* in the *LRRK2* gene may or may not display LBs, and those with other *LRRK2* mutations often lack LBs despite significant degeneration of dopamine neurons in the SNpc (Schneider and Alcalay 2017; Kalia et al. 2015). Similarly, PD patients with the PARK2 mutations show sparsely distributed LBs with unique patterns compared to idiopathic PD cases (Doherty et al. 2013).

Both dementia with Parkinson's disease dementia (PDD) and Lewy bodies (DLB) possess common characteristics seen in LB dementias, including impairment of cognitive performance, parkinsonism (a requirement in PDD, though not consistently observed in DLB), fluctuations in cognitive function and alertness, along with visual hallucinations, which are key features. REM sleep behavior disorder has currently been identified as an essential feature of DLB and frequently occurs in PDD (McKeith et al. 2017; Donaghy et al. 2015).

Distinguishing PDD from DLB relies on the “1‐year rule,” which associates dementia onset after 1 year of parkinsonism with PDD and simultaneous or preceding dementia within a year with DLB (Prasad et al. 2023; Borghammer et al. 2024). Despite criticisms of its arbitrariness, this rule proves clinically useful. Numerous studies employing this criterion suggest that DLB tends to be more severe than PDD, particularly in cognitive and potentially neuropsychiatric domains (Bougea et al. 2018). Differentiating between PDD and DLB based on a single examination can be challenging or even impossible, especially without considering the time‐based progression of events. As the disease progresses, similar features of the two conditions grow more pronounced, resembling patterns seen in many neurodegenerative disorders. Additionally, the influence of other comorbidities increases over time. A recent Swedish study found that mortality rates for PDD and DLB are similar, both about three times greater than those of the general population, highlighting the severity of these conditions (Larsson et al. 2018).

Despite variations in the order of clinical symptoms that develop in PD, PDD, and DLB, they exhibit the common neuropathological feature considered as a key factor in PD motor symptoms, as well as extrapyramidal symptoms along with cognitive impairment in both DLB and PDD (Braak et al. 2003; Spillantini et al. 1998; Mori 2005; Hurtig et al. 2000; Tsuboi et al. 2007; Irwin et al. 2012). The pathological differentiation between PD and PDD/DLB centers on the advancement of disease pathology across the brain. LB inclusions in PD are primarily found in the limbic regions and brainstem, whereas in DLB and PDD, they reach the neocortex. Distinguishing between PDD and DLB based on postmortem neuropathological observations is challenging due to shared common pathologies (McKeith et al. 2017; Braak et al. 2003).

## 3 Epigenetics in Cognition and PD

Epigenetics has a crucial role in regulating phenotypic plasticity, enabling organisms to dynamically adjust their traits and characteristics as a reaction to environmental stimuli and other factors (Huynh and Casaccia 2013). Epigenetics is one of the principal mechanisms in the process of cell differentiation, where cells within the same species, despite having identical DNA, acquire diverse appearances and functions (Felsenfeld 2014).

Epigenetics includes three primary categories: (1) histone modifications, (2) microRNAs (miRNAs), and (3) DNA methylation, all of which will be explored in detail. Exact regulation of these mechanisms is necessary for preserving regular cellular function. However, when these mechanisms are dysregulated or disrupted, they can be factors in the development of various illnesses (Aslani et al. 2017).

Epigenetic processes are integral to maintaining physical health, preserving mental well‐being, and sustaining cognitive abilities. They have a vital function in memory formation, cognitive functions, and learning by combining genetics to environmental influences. For example, maternal behavior in rats has been shown to induce changes in the HPA axis along with expression of hippocampal glucocorticoid receptor in the progeny, impacting stress responses and persisting into adulthood through epigenetic regulation, including histone acetylation and DNA methylation. This research highlights the significance of epigenetic regulation in shaping cognitive outcomes (Weaver 2007).

Epigenetic processes, including histone 4 acetylation, control gene expression throughout the extinction of conditioned fear, which is essential for managing cognitive behavioral impairments in patients with anxiety disorders. These mechanisms enhance the expression of brain‐derived neurotrophic factor (*BDNF)* and contribute to the reduction of conditioned fear through the enhancement of long‐term memory formation and synaptic plasticity (Bredy et al. 2007).

A study by Miller and Sweatt (2007) revealed the participation of epigenetic processes in the formation of memories in rats. These results demonstrated that encountering fear‐inducing stimuli led to increased DNA methyltransferase (DNMT) expression, causing heightened methylation that downregulated the memory‐suppressing gene PP1. Moreover, the same triggers induced demethylation and upregulated the reelin gene, essential for promoting synaptic plasticity.

Moreover, disturbances in epigenetic mechanisms have been identified as underlying factors in various cognitive disorders, including AD, drug addiction, schizophrenia, Rett syndrome, and autism spectrum disorders (Day and Sweatt 2011).

The study of epigenetics has revolutionized our knowledge of cognitive processes, offering valuable insights into how environmental factors impact cognition and opening new avenues for targeted interventions in cognitive disorders. This knowledge has great potential for addressing and correcting the effects of environmental influences on cognition, as well as for developing more specific and effective treatments for cognitive disorders.

As of 2019, PD was believed to affect more than 8.5 million people globally (WHO 2022). A meta‐analysis demonstrated that PD incidence rises with age in both males and females, with a more significant rise among men in the 60–69 and 70–79 age groups (Hirsch et al. 2016). Common clinical presentations include a movement‐related disease distinguished by resting tremor, bradykinesia, and rigidity, while postural instability typically appears at a higher stage (Cacabelos 2017). Several risk factors—including smoking, caffeine intake, and pesticide exposure—are thought to affect PD development, though the underlying mechanisms remain unclear (Cacabelos 2017).

While PD is predominantly considered an idiopathic condition, nearly 10%–15% of reported patients show a family history, and about 5% exhibit Mendelian inheritance patterns (Deng et al. 2018). A total of 23 PARK genes have been related to PD, exhibiting autosomal dominant or recessive patterns of inheritance (Schulte and Gasser 2011). The GBA1 gene mutations, which encode β‐glucocerebrosidase and are associated with Gaucher disease, are a crucial genetic risk factor in PD (Sidransky and Lopez 2012). Additional genetic risk factors include the *MAPT* gene, the major histocompatibility complex, and class II (HLA‐DQB1) (Nalls et al. 2014), responsible for encoding tau along with additional proteins (Simón‐Sánchez et al. 2009).

Studies exploring the involvement of epigenetics in PD are summarized as follows. H. Song et al. (2023) uncovered various epigenetic processes, like DNA methylation and RNA‐mediated pathways, chromatin remodeling, and posttranslational histone modifications that are involved in PD progression. In separate research, Goers et al. (2003) discovered that higher histone acetylation levels on *H2A*, *H3*, and *H4* were observed in dopaminergic neurons extracted from PD cases versus healthy controls, indicating substantial chromatin remodeling in PD.

Also, miRNAs serve a crucial function in PD pathogenesis (Mouradian 2012). For example, miR‐153 and miR‐7 are responsible for downregulating the SNCA gene, which is implicated in α‐Syn accumulation and death of neuronal cell. Decreased expression of these miRNAs has been recognized as a candidate factor for PD diagnosis, particularly in brain areas related to the disease's neuropathology, like the SNpc (McMillan et al. 2017; Doxakis 2010).

Leukocytes isolated from PD patients' peripheral blood have shown reduced methylation levels in specific CpG‐2 sites of the *SNCA* gene promoter compared to the control group, highlighting their potential role as PD biomarkers. Additionally, methylation‐mediated inhibition of a CpG island within intron 1 of the SNCA gene resulted in upregulated SNCA expression in the cortex and putamen of PD patients, indicating a hypomethylation pattern in these areas. The collected data highlight the significance of DNA methylation in PD pathogenesis and its possible application as a biomarker (Jowaed et al. 2010; Tan et al. 2014; de Boni et al. 2015).

It is hypothesized that epigenetic components involved in PD pathogenesis may also contribute to the development of cognitive deficits, suggesting a potential but as yet unconfirmed association between epigenetic dysregulation and cognitive decline in PD patients.

### 3.1 Epigenetics in Cognitive Dysfunction

Current evidence remains insufficient to define the role of epigenetic processes in the cognitive and memory impairments observed in PD patients. We therefore propose that identifying and investigating this possible link could guide future studies and may ultimately open new therapeutic avenues.

## 4 DNA Methylation

Among the various epigenetic alterations studied in individuals with AD, DNA methylation stands out as an investigated mechanism. This process involves the modulation of gene expression by adding a methyl group to cytosine at the C5 position. DNMT enzymes play a crucial part in facilitating this process by transferring the methyl group from S‐adenosylmethionine to the fifth carbon atom in the cytosine base. Within the category of DNMT enzymes, there are distinct types, each possessing unique characteristics. One such type is the de novo category, which includes enzymes Dnmt3a and Dnmt3b, which contribute to the creation of unique methylation patterns. Another type is the conventional category, which includes the Dnmt1 enzyme, whose role involves transferring the existing methylation pattern from the maternal DNA to the daughter DNAs during cell division and DNA replication processes (Moore et al. 2013).

In mammals, CpG islands—regions of DNA spanning approximately 1000 base pairs—are of substantial importance. These islands are characterized by a high density of CpG sites encompassing around 70% of gene promoters (Saxonov et al. 2006). Methylation within these promoter areas may inhibit gene expression, playing a key role in gene imprinting (Moore et al. 2013).

**FIGURE 2:**
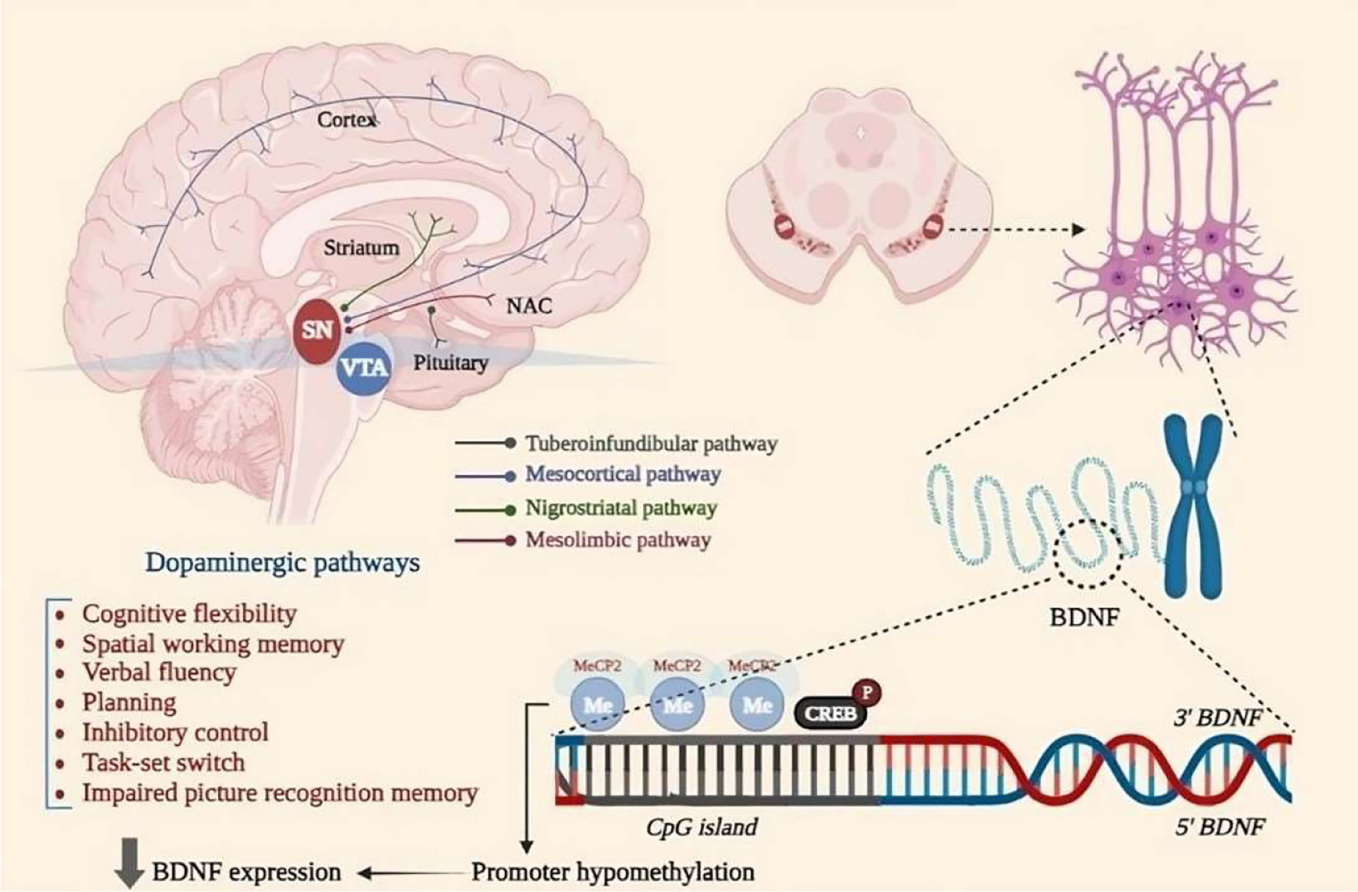
The possible role of DNA methylation in cognitive impairment seen in PD patients.

Several investigations have examined how DNA methylation correlates with cognitive processes, with specific emphasis on synaptic plasticity, which directs to the capability of neurons to undergo structural and functional alterations along with establishing new connections in response to environmental stimuli and sensory input. Feng et al. investigated the effects of the loss of the DNA methyltransferases Dnmt1 and Dnmt3a on hippocampal neuronal plasticity. They found that deletion of the genes encoding these enzymes in forebrain excitatory neurons impaired neuronal plasticity, leading to deficits in learning and memory (Feng et al. 2010). Figure 2 summarizes the data that have been hypothesized to indicate a possible role of DNA methylation in cognitive impairment in PD patients

Several studies have been performed to assess the epigenetic modifications in PD (H. Song et al. 2023; Pavlou and Outeiro 2017).

DNA methylation, an epigenetic modification, is catalyzed by DNMTs, which add a methyl group to cytosine, transforming it to 5‐methylcytosine (5‐mC) at cytosines adjacent to guanines, known as CpG islands (H. Song et al. 2023). Although hypermethylation of certain genes silences gene expression, hypomethylation of DNA can lead to increased gene expression. Masliah et al. (2013) examined DNA methylation patterns over the entire genome in samples obtained from the brains and blood of PD patients, which identified methylation patterns in genes previously known to be related to PD, hence supporting the potential role of epigenetic alterations as a molecular mechanism for PD.

In an epigenome‐wide association study (EWAS) conducted by Masliah et al. on 232 PD patients to analyze methylation markers, a decline in cognitive function (by losing ≥ 4 MMSE points) was associated with 7 CpGs, which are located in KCNB1, DLEU2, and SATB1 genes (Masliah et al. 2013).

Although direct evidence linking DNA methylation to cognitive impairment in PD patients remains limited, we propose three hypotheses—based on current literature—that may explain potential mechanisms.

The initial hypothesis centers on the dysregulated expression of BDNF. The role of BDNF encompasses supporting neuron growth, sustaining their viability, and enhancing synaptic plasticity. It is also essential for activity‐dependent neuroplasticity, establishing the foundation for memory and learning in the hippocampus (Y. Wang et al. 2016). Different neurodegenerative conditions, like PD, AD, and Huntington's disease, have been linked to a reduction in BDNF expression (Habibi et al. 2011). DNA methylation and other epigenetic modifications have an influence on modulating BDNF expression (Martinowich et al. 2003). Howells et al. observed robust of BDNF mRNA expression in the SNpc via in situ hybridization in the study, with a 70% reduction noted in clinically and neuropathologically defined PD. This reduction is believed to be partly owing to the loss of dopaminergic neurons; however, a 20% decrease in expression of BDNF mRNA was also observed in the surviving dopaminergic neurons of the SNpc in PD patients in comparison with healthy controls (Howells et al. 2000). Muñoz et al. (2010) identified a correlation between enhanced outcomes of the novel object recognition task and increased BDNF expression, accompanied by alterations in DNA methylation at the BDNF promoter I region in the mice hippocampus (Muñoz et al. 2010). A correlation was identified between the enhancement of fear‐based learning and increased expression of BDNF exon IV, while presentation of the context by itself was associated with elevated levels of BDNF exons I and VI. These results suggest differential engagement of hippocampus genetic segments as a result of various forms of cognitive engagement, such as the acquisition of environmental information as opposed to emotional contextualization of the same environment. Interestingly, the results indicate that increased expression of transcripts containing BDNF exon IV corresponds with reduced DNA methylation at the corresponding initiator of gene expression. This decline correlated strongly with higher hippocampal BDNF mRNA expression— particularly exon IX—during the consolidation of fear‐associated memories (Lubin et al. 2008).

Moreover, according to a 2016 study, a decrease in BDNF levels is strongly linked to impaired cognitive performance in individuals with PD (Y. Wang et al. 2016). Examining BDNF‐related epigenetic alterations may uncover a common underlying pathology.

The second hypothesis involves peroxisome proliferator‐activated receptor gamma coactivator−1 α (PGC−1α). The correlation between mitochondrial dysfunction and PD has been investigated in numerous studies. Serving as a coactivator of nuclear receptors, this gene is a key regulator of energy metabolism, transcription, mitochondrial activity, and microglial adaptability (Yang et al. 2020).

Methylation changes in the PGC‐1α gene within the substantia nigra of PD patients were investigated by Su et al. Hypermethylation and suppression of *PGC‐1α* were seen in this study (Su et al. 2015).

Conversely, multiple studies have highlighted a possible correlation between PGC‐1α and cognitive dysfunction (Gong et al. 2013; Zhao et al. 2021; B. Han et al. 2020). As evidence, B. Han et al. (2020) reported that upregulation of PGC‐1α alleviated cognitive deficits by lowering reactive oxygen species (ROS) accumulation. Even though there are limited data linking PGC‐1α gene methylation to thinking and memory, the positive effects of increasing its activity deserve more research.

The third hypothesis involves the MAPT gene. Abnormal aggregation and inclusion formation of the microtubule‐associated protein tau (MAPT) are key manifestations of neurodegenerative disease, like PD. Due to this common pathology, these disorders are commonly referred to as tauopathies (B. Han et al. 2020). Coupland et al. (2014) reported that MAPT hypermethylation across various brain regions may serve as a neuroprotective compensatory response during disease onset or progression. *MAPT* gene has two haplotypes of which H1 is correlated with the risk of developing PD and AD (Allen et al. 2014; Simón‐Sánchez et al. 2009). Reduced 3R.tau to 4R‐tau and considerably lower soluble tau in SN of PD subjects also approve the similar tauopathy among PD and AD patients (Duan et al. 2013; X.‐A. Liu et al. 2012).

Furthermore, the substantial effects of MAPT on cognition and dementia risk have been demonstrated in multiple studies (Setó‐Salvia et al. 2011; Winder‐Rhodes et al. 2015). As reported by Tunold et al. (2021), strong links were identified between dementia onset and the coexistence of MAPT H1 and APOE ε4 haplotype in PD patients.

In a longitudinal research (4 years) by Jiskoot et al. (2018), presymptomatic carriers of MAPT and GRN mutations were followed alongside healthy controls, revealing significant cognitive declines—particularly in language, attention, executive function, and social cognition—among MAPT mutation converters. Winder‐Rhodes et al. (2015) reported that in PD patients, there is a correlation between MAPT homozygosity and picture recognition memory.

Nonetheless, research investigating the link between MAPT gene methylation or other epigenetic mechanisms and cognition remains scarce, and this association should therefore be regarded as a hypothesis requiring further validation.

## 5 Epigenetic Modifications

Several studies have been performed to review the epigenetic modifications in PD (H. Song et al. 2023; Pavlou and Outeiro 2017). DNA methylation is one of these modifications, performed by DNMTs. This enzyme mediates the addition of a methyl group to cytosine, converting it to 5‐mC adjacent to guanines, forming the CpG islands (H. Song et al. 2023). Gene hypermethylation generally suppresses expression, while hypomethylation tends to enhance it. A study conducted by Masliah et al. assessed the patterns of genome‐wide DNA methylation in samples obtained from the brains and blood of individuals diagnosed with PD, identifying methylation patterns in genes whose association with PD was established. These findings reinforce the contributions of epigenetic modifications in the underlying PD pathology (Masliah et al. 2013). DNA methylation has also been a targeted promising therapy addressing cognitive decline in PD. Compounds such as 5‐aza‐2′‐deoxycytidine and RG108 have been assessed in the treatment of PD. These factors inhibit DNMTs, induce DNA demethylation, and reverse PD‐associated methylation (Schneider et al. 2020). Y. Wang, Wang, et al. (2013) demonstrated that a DNMT inhibitor called 5‐aza‐2′‐deoxycytidine increased α‐synuclein and tyrosine hydroxylase dopamine production via CpG demethylation in the promoter of the α‐synuclein gene. Nevertheless, issues associated to stability and bioavailability of RG108, and bone marrow suppression and gastrointestinal side effects of 5‐aza‐2′‐deoxycytidine affecting their efficacy remain unanswered (Eldin et al. 2023).

## 6 Histone Modification

Histones are essential for nuclear DNA organization, primarily through the formation of nucleosomes, the core structural components of chromatin. Every single nucleosome consists of 147 base pairs (bp) of DNA wrapped around a histone octamer, which includes a pair of each core histone: H2A, H2B, H3, and H4 (Pal and Tyler 2016; Levenson and Sweatt 2005). The H1 histone serves as a connector, stabilizing the chromatin structure by connecting adjacent nucleosome units, thereby enabling the compact packaging of DNA (Höllmüller et al. 2021).

**FIGURE 3:**
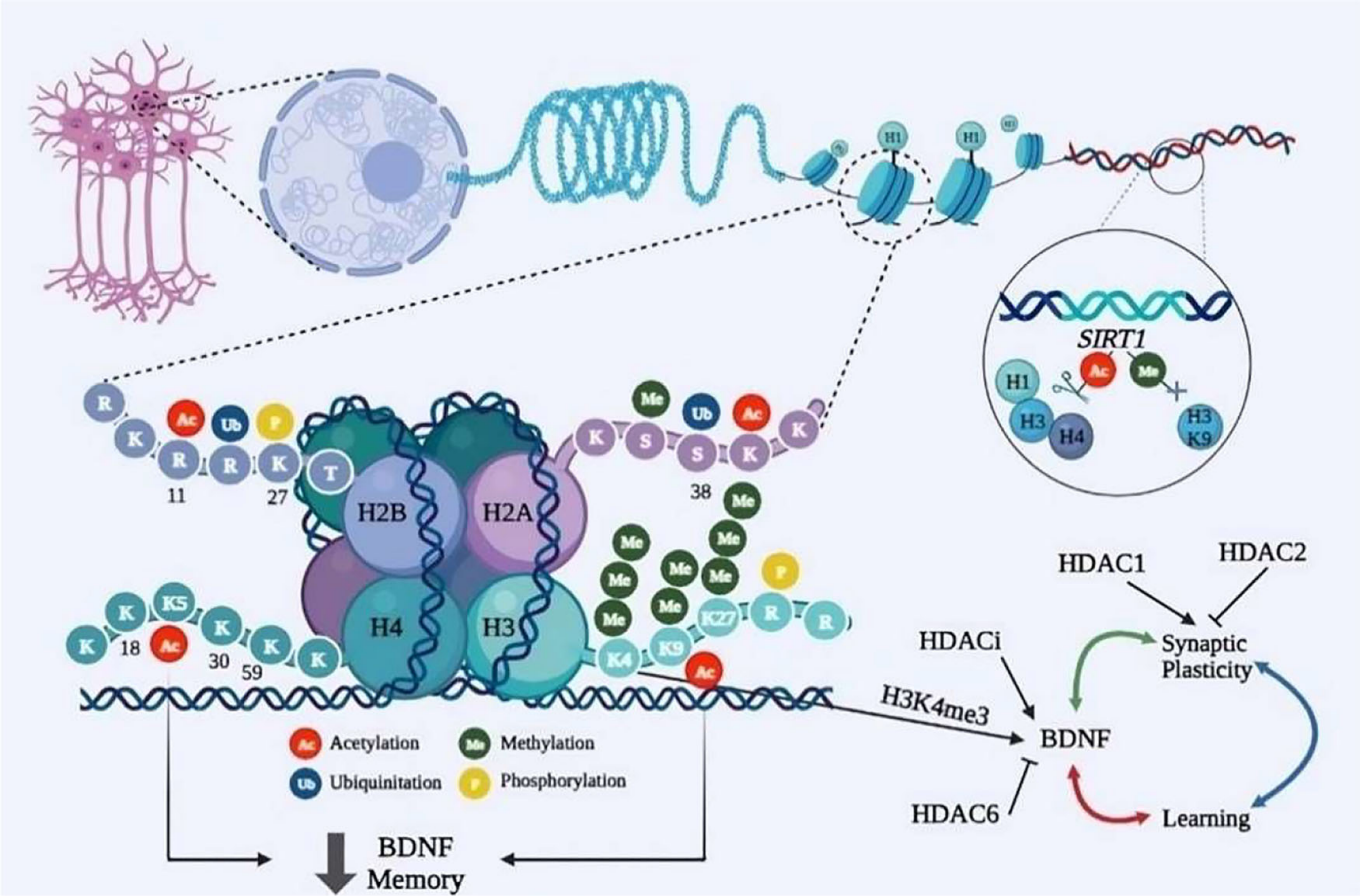
The nucleosome's structure and potential epigenetic pathways implicated in cogitation processes in PD.

Figure 3 presents the structure of nucleosomes and outlines potential epigenetic pathways associated with cognition in PD.

Methylation, acetylation, phosphorylation, ubiquitylation, and SUMOylation occur primarily in the N‐ histones terminal tails. These modifications profoundly affect chromatin structure and control the availability of genetic data in the nucleus, playing as central regulators of gene expression and enabling cellular responses to internal and external cues (Levenson and Sweatt 2005; Hwang et al. 2017).

Extensive research in animal models has explored how histone acetylation contributes to learning, synaptic plasticity, and memory formation. In a study by Lubin et al., contextual fear conditioning in adult mice led to heightened acetylation of histone H3 at the BDNF promoter within the hippocampus, coinciding with learning and memory impairments. This finding underscores the importance of histone acetylation in cognitive mechanisms and supports the hypothesis that it may influence memory formation (Lubin et al. 2008). Histone acetylation is elevated in NF‐κB‐targeted gene regions involved in the process of memory consolidation (Federman et al. 2013; de la Fuente et al. 2015).

The functions of posttranslational modifications (PTMs) can differ according to the cell type, highlighting the high specificity of cell‐dependent epigenetic changes. For instance, in oligodendrocytes, histone acetylation negatively affects myelin formation by activating genes that inhibit myelin production. Conversely, in hippocampal neurons, histone acetylation is essential for learning and memory functions, counteracting age‐related cognitive decline. Proper myelin development requires the suppression of transcriptional inhibitors of myelin genes, and natural deacetylation processes are essential for repairing defective myelin—a process that unfortunately diminishes with age.

### 6.1 Dopaminergic Neurons and Histone Modifications in PD

Depletion of dopaminergic neurons within the substantia nigra is a central characteristic of PD pathology. The regulation of the *SNCA* gene, which encodes α‐Syn, has been specifically correlated with histone modifications, such as methylation of histone H3 at lysine 4 (H3K4me3) and lysine 27 (H3K27me3). Elevated H3K4me3 levels at the SNCA promoter have been observed in postmortem PD brain tissue, whereas neurotoxin‐treated PD cell cultures demonstrate a reduction in both H3K4me3 and H3K27me3. The results suggest a possible role for altered histone modifications in regulating gene expression, including α‐Syn, although this remains to be confirmed by further studies. Nevertheless, additional research is necessary to elucidate the role of distinct histone methylation patterns in modulating gene expression and contributing to PD pathogenesis (Basavarajappa and Subbanna 2021; Chatterjee et al. 2017).

### 6.2 Histone Modifications in Neurodegenerative Diseases and Cognitive Impairment

The following discussion outlines key histone modifications and their gene regulatory impacts on molecular pathways, especially in the context of PD and cognitive impairments.

Histone H3 lysine 9 dimethylation (H3K9me2) is a modification involved in both memory formation and PD. H3K9me2 is known to suppress genes participating in the memory process, and memory impairments may be induced by disruptions in this epigenetic mechanism (Morse et al. 2015). The study by Gupta‐Agarwal et al. demonstrated that higher H3K9me2 in the lateral amygdala strengthened memory consolidation. Similarly, rats showed elevated H3K9me2 in the hippocampal CA1 region and entorhinal cortex during memory consolidation (Gupta‐Agarwal 2014).

The impact of α‐Syn on chromatin and histone adjustments was explored in several studies (Riddle et al. 2011). In transgenic Drosophila and inducible SH‐SY5Y neuroblastoma cells, α‐Syn overexpression resulted in elevated levels of histone H3K9 methylation, particularly mono‐ (H3K9me1) and di‐methylation (H3K9me2). The upregulation of H3K9me2 was correlated with alterations in the expression of L1CAM‐encoding genes involved in cellular adhesion (Riddle et al. 2011). Typically linked to the suppression of gene expression, H3K9me2 contributes to processes of memory formation and neuronal plasticity (Satake et al. 2009). The results also highlighted the involvement of euchromatic histone‐lysine N‐methyltransferase 2 (EHMT2) in catalyzing H3K9me2 generation, underscoring its capability to be involved in PD‐related processes. These results suggest that *H3K9* demethylation contributes to the epigenetic regulation underlying PD pathogenesis.

In contrast, some research indicates that H3K9me2 might contribute to cognitive processes. Gupta et al. (2010) observed that exposure to a fear‐inducing environment triggered both H3K4 trimethylation and H3K9 demethylation in the hippocampus, suggesting dual regulation of gene expression (both upregulation and downregulation) in memory formation. Interestingly, inhibition of histone deacetylases (HDACs) using sodium butyrate reduced *H3K9* demethylation, potentially enhancing memory processes. Furthermore, an increase in H3K9 demethylation was noted in the hippocampus CA1 subregion after subjects underwent fear conditioning and encountered a new setting. These findings suggest a potential role for H3K9 demethylation in memory processes triggered by associative learning and exposure to novel environments, a hypothesis that warrants further validation.

### 6.3 Histone Modifications in PD and Cognitive Impairment

Research demonstrates elevated H3K9 demethylation in the hippocampus CA1 region after both novel environment encounter and fear conditioning, underscoring its involvement in associative and context‐specific memory processes.

#### 6.3.1 H3K4me3 and H3K27me3

Histone methylation can modulate gene expression, with *H3K4* trimethylation (H3K4me3) typically promoting transcription and *H3K27* trimethylation (H3K27me3) often leading to transcriptional repression. H3K4me3 has been implicated in regulating iron metabolism, and epigenetic changes like those induced by 6‐hydroxydopamine (6‐OHDA) may contribute to PD pathogenesis. It was found that 6‐OHDA notably lowered H3K4me3 and H3K27me3 concentrations in the striatum. The results indicate a possible involvement of histone methylation in the advancement of PD, though the regional differences are still poorly defined (Södersten et al. 2014; Gibney and Nolan 2010).

#### 6.3.2 GSK‐J4 and Its Therapeutic Potential for PD

The histone demethylase inhibitor GSK‐J4, which effectively traverses to the blood–brain barrier, has demonstrated strong potential in lowering labile iron within dopaminergic neurons and reducing cell death caused by oxidative stress. In 6‐OHDA‐induced PD models, GSK‐J4 restored normal concentrations of H3K27me3 and H3K4me3, with its neuroprotective effects primarily attributed to H3K4me3 regulation. These results suggest that GSK‐J4 has potential therapeutic benefits compared to traditional iron chelators, although this remains to be validated in further studies (Mu et al. 2020).

#### 6.3.3 H3K4me3 and Its Role in PD and Memory Impairment

H3K4 trimethylation (H3K4me3) is a transcriptional activation marker associated with memory processes. Research examining histone modifications in the SNCA gene's regulatory sequences revealed increased levels of H3K4me3 at the SNCA promoter in postmortem PD tissues, neuronal cell models, and PD‐derived induced pluripotent stem cells. Decreasing H3K4me3 levels led to a decrease in α‐synuclein expression, suggesting a potential therapeutic strategy for lowering α‐synuclein levels in PD. The study proposed using JARID1A to decrease H3K4me3 at the *SNCA* promoter, efficiently reducing α‐synuclein expression (Mullen et al. 1992; Guhathakurta et al. 2021).

The presence of H3K4me3 marks is linked to BDNF expression, which is vital for supporting synaptic plasticity and strengthening memory. In a study by Morse et al. (2015), histone lysine methylation was correlated with memory formation and learning. In the hippocampus, learning was linked to enhanced transcription of BDNF and a parallel rise in H3K4me3 at the BDNF promoter. The research found that aged hippocampi exhibited elevated baseline levels of H3K4me3 and H3K9me2, causing a disrupted equilibrium of methylation and histone acetylation and contributing to memory impairment. These observations in PD patients imply that alterations in H3K4me3 levels may contribute to memory deficits, a possibility that requires further confirmation (Srivastav et al. 2018).

#### 6.3.4 H3K27me3 in PD Pathogenesis

The chromatin repressor H3K27me3 has an essential role in modulating the expression of the SNCA gene. In the substantia nigra of the adult human brain, H3K27me3, along with H3K4me3 and H3K27ac, was enriched at the *SNCA* regulatory regions. H3K4me3 and H3K27ac serve as markers for transcription initiation and enhancer regions, correspondingly, but H3K27me3 is linked to silencing gene activity. These epigenetic alterations point to H3K27me3 as a regulator of SNCA, where its dysregulation could contribute to PD pathogenesis and other synuclein‐related disorders (Guhathakurta et al. 2021, 2017).

Removal of H3K27me2/3 by the demethylase UTX is critical for the process of gene activation. Tang et al. demonstrated that UTX deficiency impairs synaptic plasticity and neuronal development, resulting in cognitive and mood disturbances in UTX conditional knockout mice. These results demonstrated the role of UTX in maintaining normal neuronal function and suggested that H3K27me3 modifications may influence neurodevelopment and cognitive processes (Tang et al. 2017).

#### 6.3.5 H3K9ac

Harrison et al. (2018) analyzed SNpc protein samples from controls and patients with early and late PD for histone acetylation levels, specifically AcH3‐Lys9. The results demonstrated increased AcH3‐Lys9 acetylation in the SNpc of PD subjects in contrast to the control group (Harrison et al. 2018). This increase was marginally detectable in early PD subjects but notably higher in late PD patients. The acetylation level correlated with the Braak stage of PD (Braak et al. 2003), indicating an association between histone acetylation and PD progression. The study additionally assessed the levels of important proteins like tyrosine hydroxylase (TH), human leukocyte antigen DP‐1 (HLA‐DP‐1), HDACs, and sirtuins (SIRTs). In PD cases, *HLA‐DP1* expression elevated, whereas TH expression diminished. At the same time, no significant temporal variation in HDAC and SIRT expression levels was found. This evidence suggests a potential involvement of histone acetylation and specific protein expression in the onset and evolution of PD.

In a separate mouse study, boosting histone lysine acetylation was shown to significantly improve memory and learning, with notable effects in fear extinction experiments. HDAC inhibitor (HDACi) treatment boosted memory function, strengthened synaptic plasticity, and supported fear extinction in mice. Collectively, the data demonstrate the translational capacity of developing therapies targeting histone acetylation for treating anxiety disorders (Hait et al. 2014).

Increased histone acetylation at H3K9 and H4K5 was observed in young control mice following contextual fear conditioning, pointing to a previously unrecognized role for acetylation in memory processes. The study identified HDACs as key regulators of this process, specifically noting that HDAC2 negatively affects memory consolidation and neuronal plasticity, whereas HDAC1 does not (Peixoto and Abel 2013).

In 2023, Huang et al. explored how microglia exhibit “trained immunity,” enabling them to recall past inflammatory exposures as part of their innate immune function. The authors used lipopolysaccharide (LPS) as a priming agent and manganese (Mn) as a subsequent environmental stressor. LPS‐exposed microglia showed an amplified inflammatory reaction upon Mn exposure. The sustained elevated immune response was linked to epigenetic reprogramming, notably through elevated levels of histone 3‐lysine 27 acetylation (H3K27ac). Similar findings were replicated in PD‐affected human brains after death and in corresponding mouse models. Inhibition of H3K27ac deposition improved mitochondrial function and reduced the inflammatory response. Overall, the study suggests that microglial memory and subsequent inflammatory responses are regulated by H3K27ac‐driven epigenetic reprogramming (Huang et al. 2023).

Histone modifications have been proposed as potential therapeutic targets for PD, though their clinical applicability remains challenging. To date, both pan‐inhibitors and selective inhibitors have been investigated. Trichostatin A (TSA), one of the promising pan‐HDAC inhibitors, showed protective effects on mitochondrial integrity and neuron survival (Zhu et al. 2014; Suo et al. 2015). TSA increased programmed cell death in PC12 cells; nonetheless, a solitary treatment session caused death of MPTP‐ or rotenone‐induced cell models of PD (Y. Wang et al. 2009; Guo et al. 2018; Du et al. 2014). Another cancer treatment‐approved HDAC inhibitor named suberoylanilide hydroxamic acid (SAHA) has also shown potential neuroprotection in PD models (Kidd and Schneider 2010; Chen et al. 2012). Selective HDAC1 and HDAC3 inhibitors also had an alleviating role in the dyskinesia caused by L‐dopa in MPTP‐lesioned marmoset (Johnston et al. 2013).

## 7 Micro‐RNAs

MiRNAs—types of RNA with intrinsic noncoding RNA structure—are crucial regulators of biological processes, modulating mRNA expression at the posttranscriptional level (He and Hannon 2004). Their important function during maturation involves controlling key biological events like synaptic plasticity, memory consolidation, and neuronal differentiation (O'Brien et al. 2018; Duffy and McCoy 2020). Dysregulated miRNAs drive various neurodegenerative disorders pathogenesis, like AD, which has prompted investigations into their potential role in AD‐related cognitive impairment (Juźwik et al. 2019). Although specific studies on miRNAs and cognitive deficits are limited, insights from epigenetic research on AD and memory formation are invaluable.

Emerging evidence suggests that miRNAs could serve as peripheral biomarkers for various dementias, including AD, vascular dementia, and PD (Zetterberg and Burnham 2019; Prabhakar et al. 2017). Also, these miRNAs are key contributors to cognitive functions, regulating neurodegenerative disease‐associated proteins (Nadim et al. 2017; Miquel et al. 2018). The underlying causes of cognitive deficits in PD remain incompletely understood (Aarsland et al. 2017). However, there is growing evidence about the epigenetics effect on cognitive decline in PD.

### 7.1 miR‐7

**FIGURE 4:**
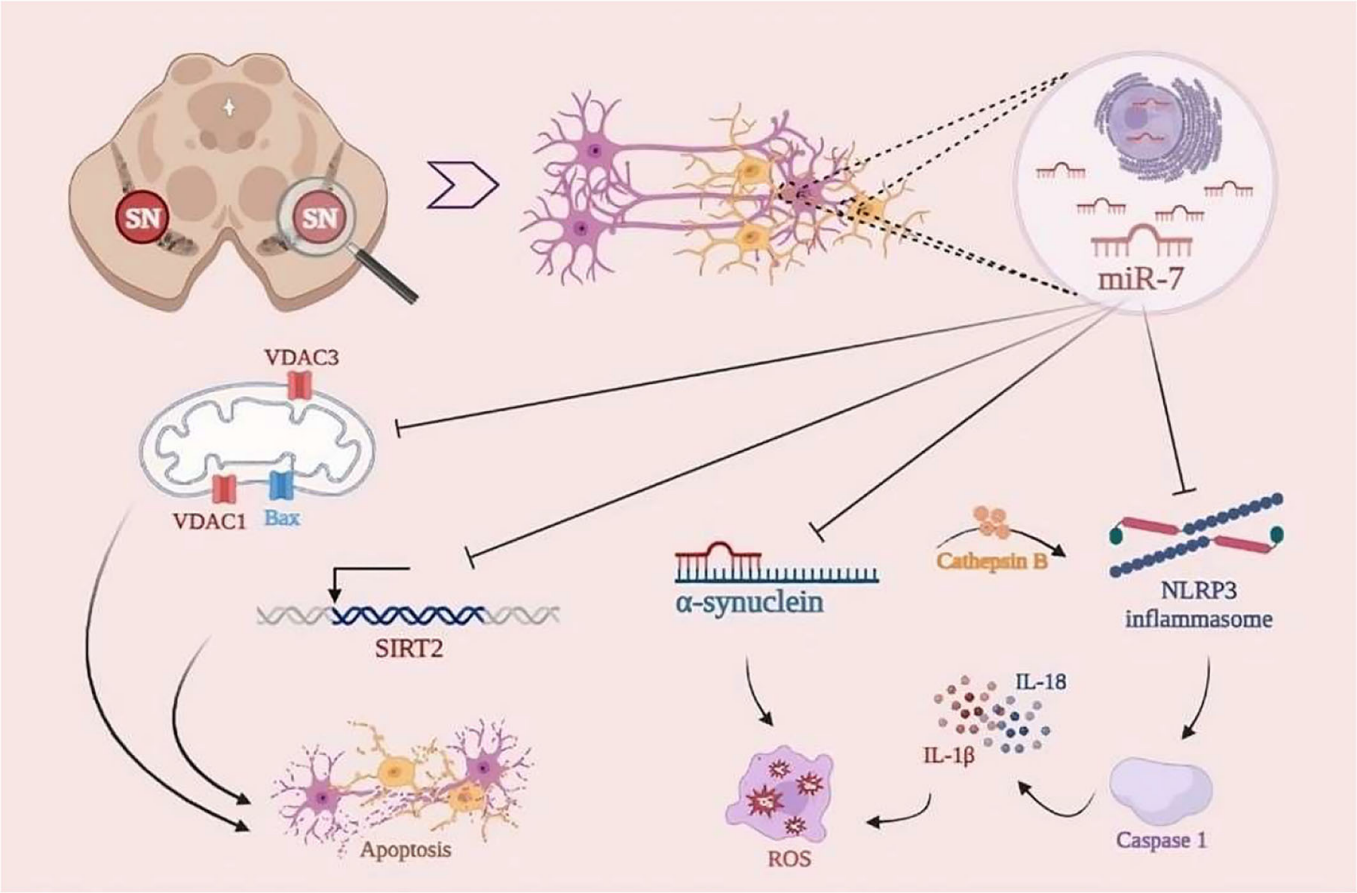
Role of mir‐7 in PD pathogenesis.

miR‐7 expression is mainly in neurons and contributes to PD pathology via base‐pairing with the 3'‐untranslated region (UTR) of α‐Syn mRNA, thereby lowering protein levels and reducing its accumulation in the brain regions most impacted, particularly dopaminergic neurons (Figure 4). The data suggest that miR‐7 holds promise as a treatment target in PD and other alpha‐synuclein disorders by strengthening cells’ inflammasome activation in microglia by targeting Nlrp3, although further studies are required to validate its therapeutic potential (Zhou et al. 2016). In PD patients, NLRP3 inflammasomes are triggered; mouse research points to α‐synuclein's impacts in modulating microglial resilience to oxidative stress (Junn et al. 2009). In Parkinson's model mice, Zhou et al. reported that miR‐7 blocks endocytosis and cathepsin B secretion. Reduced caspase‐1 expression was linked to less microglial activation and suppressed interleukin‐1β synthesis, which protected dopaminergic neurons. These results highlighted that miR‐7 and NLRP3 inflammasomes may be considered promising candidates for therapeutic intervention (Zhou et al. 2016).

In rodent ischemic models, miR‐7 mediates neuroprotection by targeting and decreasing VDAC1 and VDAC3 levels. The elevated miR‐7 expression in patients with acute ischemic stroke indicates its therapeutic relevance, as inhibiting miR‐7 has been shown to protect neurons and enhance neurological recovery following ischemia (Yao et al. 2018). Li et al. demonstrated that miR‐7 suppresses apoptosis using the human DA neuroblastoma cell line SH‐SY5Y as a PD model by targeting Bax and Sirt2 directly in a cellular PD model (Li et al. 2016).

### 7.2 miR‐124‐3p

In an in vitro study, miR‐124‐3p was found by Geng et al. to exert neuroprotective actions through targeting STAT3. Ectopic miR‐124‐3p overexpression alleviates neuronal damage, inflammation, cell death, and oxidative stress caused by MPP+, highlighting its possible role as a treatment for PD (Geng et al. 2017). Reduced expression of miR‐124‐3p was observed in a perioperative neurocognitive disorder (PND) rat model after undergoing cardiopulmonary bypass (CPB) in another study. The upregulation of this molecule exhibited protective effects against cognitive impairment, apoptosis, and inflammation in PND. The data indicate that targeting miR‐124‐3p may suggest a viable treatment approach for PND (J. Han et al. 2021).

### 7.3 miR‐124

The decreased expression of miR‐124 in PD induced the creation of miR‐124‐loaded nanoparticles that mitigated neuroinflammation by lowering pro‐inflammatory signals and cytokine mRNA levels. These changes enhance neuroprotection and decrease apoptosis, highlighting its therapeutic potential for PD (Gan et al. 2019). In PD patients, miR‐124 downregulation within the prefrontal cortex has been associated with NF‐κB signaling activation, offering new therapeutic targets (Xing et al. 2020). Pantano et al. discovered miR‐124 downregulation in the amygdala in PD, identifying modified sRNA patterns during the premotor phase of PD by deep sequencing analysis of postmortem brain samples. These findings suggest early pathogenic changes and the utility of this tool for exploring sRNA expression patterns in various biological conditions (Pantano et al. 2016). In mutant mice, Dicer1 mutation‐induced miR‐124 downregulation in hippocampal neurons was associated with better memory performance, suggesting miR‐124's key role in cognitive functions (Konopka et al. 2010).

### 7.4 miR‐195

miR‐195 dysregulation is associated with PD, where its downregulation in LPS‐stimulated BV2 cells results in microglia activation and pro‐inflammatory cytokine secretion. By inhibiting pro‐inflammatory and enhancing anti‐inflammatory cytokines, miR‐195 overexpression shows potential therapeutic effects in managing PD‐related neuroinflammation (Ren et al. 2019).

### 7.5 miR‐190

miR‐190 downregulation in PD is associated with triggering Nlrp3‐mediated neuroinflammation and neuronal damage in mice. Inflammation and neuronal damage were attenuated by miR‐190 overexpression, pointing to its possible therapeutic application in PD (Sun et al. 2019). miR‐190 may exert dual functions in cognitive processes associated with PD, carrying therapeutic potential for modulating neuroinflammation and neuronal injury, and postoperative cognitive dysfunction (POCD).

### 7.6 miR‐146a

miR‐146a's regulation of GDNF impacts glial cell activity, which has the capability to contribute to the progression of PD (Kondratyev and Gale 2000), and additionally, levels of miR‐146a decreased in PD patients in contrast to healthy individuals (Caggiu et al. 2018).

### 7.7 miR‐155

miR‐155 upregulation in the hippocampus was demonstrated in an AD rat model, coinciding with higher inflammatory cytokine levels. miR‐155 inhibition reduced inflammation and improved learning performance, pointing to its role in regulating memory impairment through neuroinflammatory mechanisms. miR‐155 is suggested as a candidate for therapeutic intervention aimed at mitigating cognitive decline in AD (D. Liu et al. 2019).

### 7.8 miR‐133b

miR‐133b expression is significantly downregulated in PD patients relative to the control group, with significant downregulation observed in both blood and brain samples of PD patients. miR‐133b may have a role in dopamine synthesis and memory, indicating a protective influence against cognitive dysfunction triggered by anesthesia (Zhang et al. 2021).

### 7.9 miR‐126

miR‐126 disrupts insulin/IGF‐1/PI3K signaling in dopamine neurons, making them more vulnerable to neurotoxins. The enhancement of neuroprotection following miR‐126 suppression implies that increased levels of miR‐126 could contribute to PD pathogenesis and associated cognitive impairments (Kim et al. 2014).

### 7.10 miR‐144

miR‐144‐3p shows persistent downregulation in the serum of drug‐naive early PD and could be a candidate and major factor involved in PD development. Its overexpression improves mitochondrial function and neuronal survival, indicating a possible therapeutic potential.

### 7.11 miR‐148b

miR‐148b downregulation in PD is linked to neurological development and apoptosis. Its overexpression in a rat model in ischemic brain regions promotes neuroprotection by suppressing the Wnt/β‐catenin pathway, suggesting its potential for stroke recovery (J. Wang et al. 2017)

### 7.12 miR‐221

The impact of miR‐221 in counteracting oxidative injury and influencing neuronal survival processes positions it as a possible biomarker and treatment target in PD (Zamanian et al. 2023)

### 7.13 miR‐199a

miR‐199a modulates autophagy in PD; it is implicated in AD through Neuritin regulation, potentially contributing to synaptic damage and cognitive impairment (D. Song et al. 2020)

### 7.14 miR‐132

miR‐132 drives temporal memory formation, as evidenced by its upregulation following trace fear conditioning and the disruption of memory acquisition observed in mice following its knockdown—highlighting its importance in hippocampus‐dependent temporal memory (R. Y. Wang, Phang, et al. 2013). miR‐132 acts as an essential activity‐dependent regulator of cognition, affecting neuronal development, dendrite growth, and spine formation in the CNS, as shown through in situ hybridization (Hansen et al. 2013). Upregulation of microRNA‐132 counteracts the cognitive damage induced by sevoflurane in rats with AD by inhibiting FOXA1 (Cong et al. 2021). By suppressing p250GAP and activating Rac pathways, miR‐132 facilitates dendritic morphogenesis and supports synaptic plasticity dependent on neuronal activity (Wayman et al. 2008)

## 8 Therapeutic Perspectives of Epigenetics in PD Cognitive Dysfunction

Several epigenetic drugs are being explored for their potential role in treating PD, including inhibitors of HDAC, DNMT, and bromodomain and extra‐terminal domain inhibitors. Over the past two decades, HDACs, a primary set of epigenetic targets, have garnered significant focus in pharmaceutical discovery. Notably, SIRT2‐specific inhibitors—a member of the HDAC family—have shown promise in PD treatment. SIRT2 inhibition has demonstrated effectiveness in rescuing α‐Syn toxicity and modifying inclusion morphology in cellular PD models. Using small interfering RNA (siRNA) to inhibit SIRT2 expression genetically has revealed protective effects against α‐Syn‐associated toxicity. Both cellular and Drosophila PD models have exhibited dopaminergic neuron protection following treatment with these inhibitors. These results reveled an association between neurodegeneration and aging, highlighting the potential role of SIRT2 inhibitors in ameliorating PD‐related cognitive impairments and motor symptoms, although further preclinical and clinical validation is needed (Outeiro et al. 2007).

Cognitive impairments in a PD rat model were alleviated following treatment with sodium butyrate, an HDAC inhibitor. This model exhibited executive function deficits, most notably during attentional set‐shifting, resembling the patterns identified in PD patients. Treatment with sodium butyrate improved attentional set formation, indicating its therapeutic potential for addressing cognitive impairments in PD (Rane et al. 2012).

According to Song et al., dieldrin, a neurotoxic pesticide related to PD, induces core histone hyperacetylation in dopaminergic neuronal cells through proteasomal dysfunction. Anacardic acid, a histone acetyltransferase (HAT) inhibitor, reduced dieldrin‐triggered histone acetylation, prevented dopaminergic cell loss, and lowered histone hyperacetylation in mouse brain areas. These results underscore the therapeutic promise of HDAC inhibitors in PD treatment (C. Song et al. 2010).

During normal learning events, a signaling pathway is activated that entails the activation of HATs and the inactivation/removal of HDACs. HATs add acetyl groups to histone tails, relaxing the nucleosome and facilitating the attachment of DNA‐binding transcription factors. Nevertheless, the conditioned response may diminish under specific circumstances. Administering an HDACi results in the hyperacetylation of histone tails, which promotes the memory formation mechanism and strengthens the response. While this basic schematic primarily focuses on histone acetylation, it is important to consider other epigenetic processes implicated in conjunction with HDACs and HATs in this mechanism.

## 9 Discussion

The disruption of epigenetic processes acts as a key molecular connection between environmental factors, genetic risks, and neurodegenerative changes observed in PD. Each mechanism discussed in this review serves as a linked part of a balanced epigenetic network in the human body, with modifications in one route having a significant impact on the rest. For instance, DNA methylation can modulate histone H3 phosphorylation by regulating enzymes such as aurora‐B kinase, while ubiquitination of H2A can inhibit this modification (Monaco et al. 2005; Joo et al. 2007). Moreover, α‐synuclein directly interacts with histones and suppresses their acetylation, leading to aberrant expression of neuroprotective genes such as BDNF (Kontopoulos et al. 2006; Jowaed et al. 2010; Zuccato and Cattaneo 2009; Hasani et al. 2025). The cross‐talk among PARP‐1 signaling, polycomb group proteins, and histone demethylases like LSD1 exemplifies the intricate integration of DNA damage responses, chromatin remodeling, and transcriptional regulation in PD pathology (Gearhart et al. 2006; Mimasu et al. 2008; Adamczyk and Kaźmierczak 2009). These interconnected mechanisms collectively drive dopaminergic neuron degeneration and contribute to the cognitive decline observed in PD. Aberrant DNA methylation of genes such as BDNF, PGC‐1α, and MAPT, alongside dysregulated histone modifications (H3K4me3, H3K9me2, H3K27me3), disrupts transcriptional balance and synaptic plasticity, leading to impaired memory and executive function. Additionally, noncoding RNAs, particularly miR‐7, miR‐124, and miR‐195, modulate inflammatory and mitochondrial pathways, further amplifying neurodegenerative cascades (Monaco et al. 2005).

After all these significant advances in decoding the epigenetic landscape of PD, major gaps remain in linking these molecular changes to cognitive dysfunction. One of the most important challenges was the heterogeneity of patient responses, which are influenced by genetic, environmental, and epigenetic factors that vary significantly across each patient. Moreover, animal models could not simulate the complexity of human disease, particularly in disease progression, comorbidities, and individual variability in treatment responses. In vivo measurement of epigenetic markers, such as DNA methylation and histone modifications, remains another significant issue (H. Song et al. 2023; Masliah et al. 2013). Additionally, differences in experimental conditions, such as the use of different animal models, cell lines, and human tissues, further complicate the translation of epigenetic findings into effective therapies. Therefore, while animal studies offer considerable insights, there is a clear need for human‐based models to better understand the role of epigenetic modifications in cognitive dysfunction of PD and develop novel therapeutic strategies.

Future studies should aim to elucidate the spatiotemporal dynamics of DNA methylation, histone modifications, and miRNA regulation across cognition‐related regions such as the hippocampus and prefrontal cortex. Integrative multi‐omics approaches, combined with targeted molecular and proteomic analyses, are essential to uncover how these epigenetic layers interact to influence neuronal and synaptic integrity. Ultimately, such comprehensive investigations could facilitate the development of mechanism‐based therapeutic strategies targeting the epigenetic underpinnings of cognitive impairment in PD. Furthermore, to overcome the current limitations in studying epigenetic changes, novel technologies such as single‐cell epigenomics, spatial transcriptomics, next‐generation sequencing (NGS), and advanced in vivo imaging are being developed for more precise and dynamic analyses (Corces et al. 2020; Georgoula et al. 2024; Goralski et al. 2024). Single‐cell epigenomics allows for high‐resolution mapping of DNA modifications at the single‐cell level, offering insights into cellular heterogeneity in PD. Spatial transcriptomics enables the study of gene expression within tissue architecture, providing a clearer comprehension of how epigenetic alterations impact specific brain regions. NGS technologies have revolutionized the depth and accuracy of genome‐wide epigenetic profiling, while advanced in vivo imaging techniques can monitor real‐time changes within living organisms. These technologies hold great promise for bridging the gap between animal models and human studies in contribution to disease progression.

## 10 Conclusions

Addressing cognitive issues in PD patients is an urgent clinical need, as cognitive deterioration markedly diminishes quality of life, and effective treatment options are limited. Impaired myelination is a hallmark characteristic of PD pathology, and emerging research implies that epigenetic processes could have a crucial part in this dysfunction. In this study, we review the current literature, emphasizing the role of epigenetics in cognition and PD.

While numerous studies have investigated cognitive dysfunction in PD patients, research particularly examining the impact of associated epigenetic mechanisms remains sparse, and available findings are often conflicting. We present hypotheses on the possible epigenetic mechanisms driving cognitive impairment in PD, grounded in the limited and fragmented existing data.

## Author Contributions

**Fatemeh Hasani**: conceptualization, investigation, methodology, writing – original draft, writing – review & editing, visualization, data curation, software, validation. **Mohammad Ebrahim Kherad**: conceptualization, investigation, visualization, writing – original draft, writing – review & editing. **Mohammad Sharifi Sarasyabi**: writing – review & editing. **Kimia Jazi**: writing – review & editing, investigation, writing – original draft. **Ali Ashkbari**: writing – review & editing, writing – original draft. **Payam Ahmadi**: writing – original draft, investigation, methodology. **Hadise Heidarpour**: writing – original draft, writing – review & editing, data curation. **Mahdi Masrour**: writing – original draft, investigation, **Sina Khamaki**: investigation, writing – original draft, **Rahem Rahmati**: validation, visualization, **Andrei Surguchov**: writing – review & editing, writing – original draft, investigation, methodology, conceptualization, data curation, supervision

## Funding

The authors have nothing to report.

## Conflicts of Interest

The authors declare no conflicts of interest.

## Data Availability

All datasets on which the conclusions of a manuscript depend are available to readers.
